# Targeting BCL2 Family in Human Myeloid Dendritic Cells: A Challenge to Cure Diseases with Chronic Inflammations Associated with Bone Loss

**DOI:** 10.1155/2013/701305

**Published:** 2013-05-22

**Authors:** Selma Olsson Åkefeldt, Mohamad Bachar Ismail, Hélène Valentin, Maurizio Aricò, Jan-Inge Henter, Christine Delprat

**Affiliations:** ^1^Childhood Cancer Research Unit, Department of Women's and Children's Health, Karolinska Institutet, Karolinska University Hospital Solna, 171 76 Stockholm, Sweden; ^2^CNRS, UMR5239, Laboratoire de Biologie Moléculaire de la Cellule, 69007 Lyon, France; ^3^Ecole Normale Supérieure de Lyon, 69007 Lyon, France; ^4^Université de Lyon, 69003 Lyon, France; ^5^Université de Lyon 1, 69622 Villeurbanne, France; ^6^Department of Pediatric Hematology Oncology, Azienda Ospedaliero-Universitaria, Meyer Children's Hospital, 50139 Florence, Italy; ^7^Institut Universitaire de France, 75005 Paris, France; ^8^CNRS 5239, Faculte De Medecine Lyon Sud, 165 Chemin du Grand Revoyet BP12, 69921 Oullins Cedex, France

## Abstract

Rheumatoid arthritis (RA) and Langerhans cell histiocytosis (LCH) are common and rare diseases, respectively. They associate myeloid cell recruitment and survival in inflammatory conditions with tissue destruction and bone resorption. Manipulating dendritic cell (DC), and, especially, regulating their half-life and fusion, is a challenge. Indeed, these myeloid cells display pathogenic roles in both diseases and may be an important source of precursors for differentiation of osteoclasts, the bone-resorbing multinucleated giant cells. We have recently documented that the proinflammatory cytokine IL-17A regulates long-term survival of DC by inducing BCL2A1 expression, in addition to the constitutive MCL1 expression. We summarize bibliography of the BCL2 family members and their therapeutic targeting, with a special emphasis on MCL1 and BCL2A1, discussing their potential impact on RA and LCH. Our recent knowledge in the survival pathway, which is activated to perform DC fusion in the presence of IL-17A, suggests that targeting MCL1 and BCL2A1 in infiltrating DC may affect the clinical outcomes in RA and LCH. The development of new therapies, interfering with MCL1 and BCL2A1 expression, to target long-term surviving inflammatory DC should be translated into preclinical studies with the aim to increase the well-being of patients with RA and LCH.

## 1. Introduction

**Figure 1:**
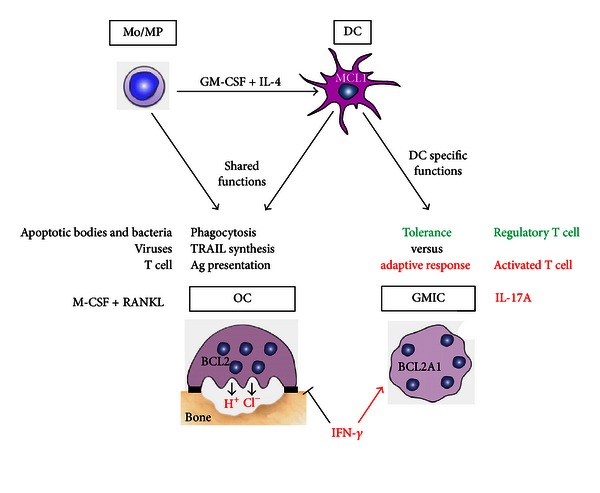
Dendritic cell functions compared to monocytes. Monocyte-derived DCs share four functions with monocytes: phagocytosis, TRAIL synthesis, Ag presentation, and differentiation into OC in the presence of M-CSF and RANKL. Conversely, initiation of tolerance, adaptive response, and IL-17A-dependent differentiation of GMIC are DC-specific functions. IFN-*γ* differentially regulates MGC formation by inhibiting OC and stimulating GMIC formation.

Myeloid dendritic cells (DCs) can be derived from monocytes in the presence of GM-CSF and IL-4, both *in vitro* and *in vivo* [1]. Therefore, DC and monocytes are closely related. They share (Figure 1) the ability to phagocytose extracellular bacteria, to synthesize the apoptotic factor TRAIL in response to interferons (IFN) in the context of viral infections [2, 3], and to present antigens to T cells. They also undergo cell fusion in the presence of M-CSF and RANKL, thus forming osteoclasts (OCs), the bone-resorbing multinucleated giant cells (MGCs) [4, 5]. By contrast, two functional properties discriminate DC from monocytes. First, DCs initiate adaptive immune responses versus tolerance, as demonstrated in mouse models of DC short-term ablation, *in vivo* [6]. Second, DCs undergo cell fusion in the presence of the pro-inflammatory cytokine IL-17A, a mechanism highly potentiated by IFN-*γ* [7, 8]. To discriminate OC from the IL-17A-dependent MGC, these latter will be called giant myeloid inflammatory cells (GMICs) in this review. Exacerbation of these fusion pathways may be involved in two diseases of unknown etiology: rheumatoid arthritis (RA) and a rare disease called Langerhans cell histiocytosis (LCH). In both diseases, bone loss is observed, and the three cytokines M-CSF, RANKL, and IL-17A have been detected [7, 9–11]. While untreated immature DCs have a short two-day lifespan, both OC and GMIC survive more than two weeks, thus demonstrating that survival pathways are activated along the DC fusion process, *in vitro*. In 2008, it was shown that B-cell lymphoma 2 (BCL2) is critical for OC survival as demonstrated by the increased bone mass of BCL2^−/−^ mice [12]. We have recently documented that an unexpected member of the BCL2 family, *BCL2A1, *is involved in the survival of GMIC [13]. In the light of this last finding, we reviewed the available knowledge to investigate whether targeting members of the BCL2 family in DC may open novel treatment opportunities in chronic inflammatory diseases associated with bone loss. We first focus on the two BCL2 members expressed by DC, MCL1, and BCL2A1. Then, we review the therapeutic compounds able to target MCL1 or BCL2A1. Finally, we discuss challenges and opportunities to target MCL1 and BCL2A1 in RA and LCH, in the aim to block formation of OC and GMIC from DC.

## 2. Bcl2 Family and the Focus on MCL1 and BCL2A1 Expressed by Myeloid Dendritic Cells

Apoptosis is initiated by extrinsic and intrinsic pathways, depending on the stimulus, as death receptor ligation on the cell surface (Fas, TNFRSF1, TNFRSF10, etc.) and intracellular stress (UV, cytokine withdrawal, cytotoxic drugs, etc.), respectively [14]. Ligation of death receptors causes formation of a death-inducing signaling complex (DISC) that triggers caspases and then apoptosis, independently of mitochondria. Such mechanism can be inhibited by CFLAR (or viral homologs), previously named FLICE-inhibitory proteins (FLIP). Interestingly, myeloid DCs expressing Fas on their membrane were not sensitive to Fas-mediated apoptosis unless sensitized by cycloheximide [15]. This resistance is attributed to the expression of CFLAR by myeloid DC. By contrast, immature (but not mature) human myeloid DCs are very sensitive to UVB irradiation [16], a process involving intracellular oxidative stress and caspase activation through the intrinsic pathway of apoptosis.

**Figure 2:**
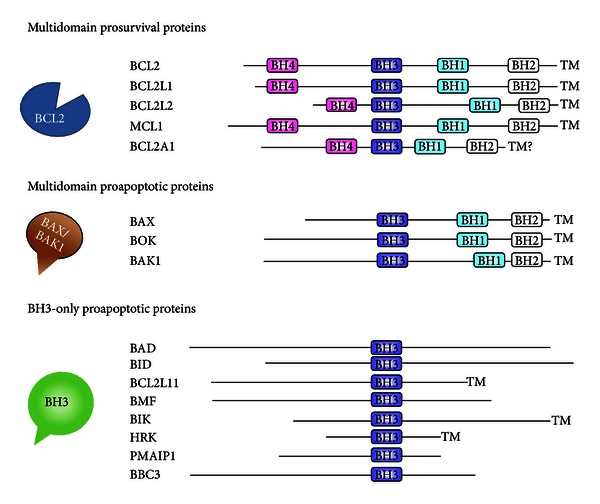
Classification of the BCL2 family into three subfamilies of BCL2-related proteins. The four BCL2 homology (BH) domains are the most highly conserved among BCL2 family. The prosurvival proteins contain four *α*-helix BH domains (BH1-4). BH1-3 domains of prosurvival proteins form a hydrophobic cleft that binds proapoptotic proteins through their hydrophobic BH3 domains.The multidomain proapoptotic proteins have BH1-3 domains, while the proapoptotic BH3-only proteins share only the BH3 domain with the other BCL2-related proteins. Most members have a carboxy-terminal hydrophobic transmembrane (TM) domain, with the exceptions of many of the BH3-only proteins and probably BCL2A1.

**Figure 3:**
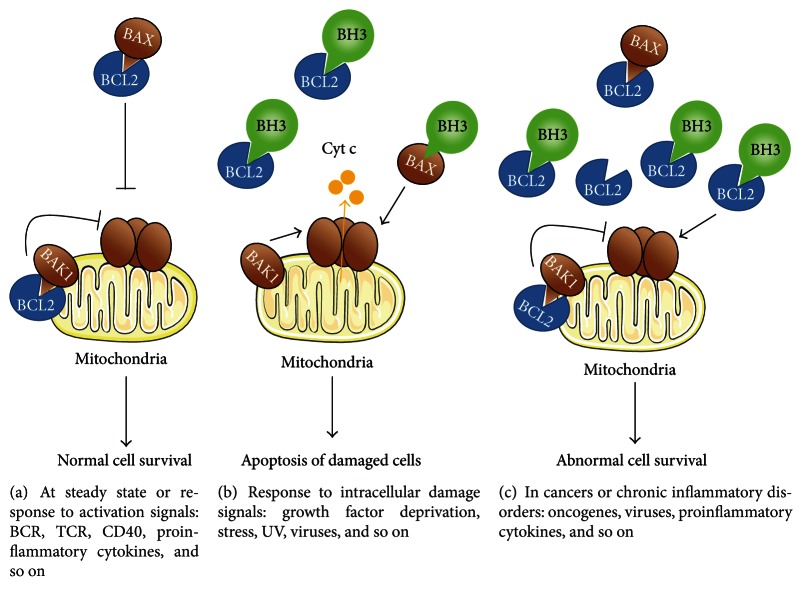
Model of survival control by the BCL2 family in physiological and inflammatory conditions. (a) In a physiological context or following an immune response, constitutive or inducible prosurvival proteins (here BCL2) bind multidomain proapoptotic proteins (BAX, BAK) that become unable to oligomerize, thereby resulting in normal cell survival at steady or activated state. (b) In response to intracellular damage, activator BH3-only proteins are induced or activated. They inhibit the prosurvival proteins and activate the effector multidomain proapoptotic proteins, which in turn are homooligomerize, triggering cytochrome c release (Cyt c) and apoptosis. Potentially abnormal cells are then eliminated. (c) In a pathological context, such as cancers and chronic inflammatory diseases, prosurvival proteins are upregulated, inhibiting the multidomain and BH3-only proapoptotic proteins. As a result, the membrane of mitochondria remains intact and abnormal cells survive.

The mitochondria-dependent intrinsic pathway involves the BCL2 members, whose main functions include embryogenesis control, tissue homeostasis, leukocyte development, and defense against pathogens. The BCL2 family displays three groups of proteins, sharing sequence homology in their BCL2 homology (BH) domains (Figure 2, Table 1). They include the prosurvival proteins (such as BCL2, MCL1, and BCL2A1) and the proapoptotic proteins, comprising the multidomain proteins (such as BAX and BAK1) and the BH3-only proteins (including BAD, BID, and BIM) [17]. How these three groups integrate cell signaling into the decision to live or die is not completely understood and the mechanism remains controversial [18]. A set of interactions between BCL2 members ultimately controls the release of cytochrome c from mitochondria, the caspase activation, and then apoptosis. In living normal cells, proapoptotic proteins are sequestered by prosurvival members, thus inhibiting the release of cytochrome c from mitochondria (Figure 3(a)). However, there are some reports indicating that the mitochondria are not essential for initiation of cell death induced from intrinsic pathway and that apoptosis can also occur in the absence of caspase activation [19]. After an intracellular apoptotic stimulus, the BH3-only proteins activate the multidomain proapoptotic proteins, thereby triggering cytochrome c release and apoptosis (Figure 3(b)). Impaired apoptosis associated with an enhanced expression of prosurvival BCL2 proteins is a hallmark of human cancers (Figure 3(c)) and is frequently associated with resistance to therapy [18, 20, 21]. In addition, upregulation of prosurvival BCL2 proteins has also been observed in chronic inflammatory disorders.

Compared to lymphocytes, relatively little knowledge is available on the regulation of myeloid cell survival by the BCL2 family members. Among the prosurvival proteins, both MCL1 and BCL2A1 appear to have physiologically important roles in regulating myeloid cell survival. MCL1 is expressed at steady state in neutrophils, monocytes, and DC, but not in macrophages, unless they are activated [21, 22]. MCL1 provides short-term enhancement of myeloid cell survival during the critical transition differentiation process [22]. In normal or tumoral myeloid cells, the cytokines GM-CSF, IFN-*α*, IL-3, IL-5, IL-6, IL-15, or IL-22 upregulate MCL1 expression [22]. Upon exposure to pro-inflammatory cytokines, MCL1 is upregulated in granulocytes, monocytes, and macrophages and associated with BCL2A1 induction [19]. During chronic inflammatory diseases, apoptosis of neutrophils is significantly delayed due to upregulation of MCL1 [23]. Interestingly, BCL2A1 is not expressed in myeloid cells at steady state; yet, inflammatory stimuli including bacterial endotoxin like lipopolysaccharide, G-CSF, GM-CSF, IL-1*β*, TNF-*α*, IFN-*γ*, IL-8, and IL-17A induce it, thus extending the survival of neutrophils, granulocytes, mast cells, macrophages, and, as we recently documented, DC [13, 19, 24–26]. BCL2A1, an NF-*κ*B target gene expressed in activated myeloid cells, supports key function in inflammation. In chronic inflammatory disorders, regulation of MCL1 and BCL2A1 gene expressions results in recruitment and stabilization of myeloid cells of the immune system [27, 28]. We propose that, in healthy DC, while MCL1 expression provides short-term (two days) survival, additional BCL2A1 expression switches the DC phenotype and allow long-term survival. In this context, BCL2A1 induction operates downstream of NF-*κ*B activation in IL-17A-stimulated DC [13] and may be very important in sustaining chronic T-cell activation in IL-17A-related diseases. This new concept places IL-17A-stimulated MCL1^+^ BCL2A1^+^ DC in the sunlight and makes MCL1 and BCL2A1 novel attractive therapeutic targets in chronic inflammatory diseases.

## 3. Targeting MCL1 and BCL2A1 by Chemotherapeutic Compounds

To overcome BCL2-family-mediated resistance to chemotherapy, different strategies have been tried, including their targeting by antisense oligonucleotides peptide inhibitors and small molecules inhibitors (SMIs) [29, 30]. Most widely used so far is the SMI ABT-737 (and its orally active follow-up ABT-263) which mimics the BH3-only proteins and binds with high-affinity BCL2, BCL2L1, and BCL2L2, inducing apoptosis in a BAX- and BAK-dependent way [31]. However, it binds only weakly to MCL1 and BCL2A1, and resistance to ABT-737 has been associated with high expression of MCL1 and BCL2A1 [32, 33].

Expression of both MCL1 and BCL2A1 has, in several hematological malignancies, been associated with chemoresistance or poor prognosis [34, 35]; thus, new drugs targeting these proteins must be developed. Some of the SMIs under development, including Obatoclax and Sabutoclax, have been shown to better target MCL1 or MCL1 and BCL2A1, respectively [36, 37]. Sorafenib, developed as a BRAF inhibitor, reduces MCL1 translation leading to increased apoptosis in leukemia cells [38] while Flavopiridol, a cyclin-dependent kinase inhibitor, suppresses MCL1 and has been used to treat patients with high-risk chronic lymphocytic leukemia (CLL) [39, 40]. 

We recently showed that monocyte-derived DCs, treated with IL-17A and IFN-*γ* that mimic chronic inflammation conditions, develop resistance to apoptosis. This resistance is associated with a robust coexpression of MCL1 and BCL2A1 and is dependent on IL-17A that induces BCL2A1 in MCL1^+^ DC [13]. IL-17A- and IFN-*γ*-treated DCs were resistant to a variety of chemotherapeutic drugs. However, they were highly sensitive to the antimicrotubule drugs vinblastine and, to a lesser extent, vincristine and cytarabine. We showed that exposure to vinblastine or cytarabine decreased MCL1 expression. Antimicrotubuli agents are widely used in various cancers, including hematological malignancies [41]. They induce mitotic arrest and trigger apoptosis through mechanisms which are not fully clear. However, Wertz et al. recently showed that vincristine-induced apoptosis is mediated by the molecular partnership between the ubiquitin-ligase FBW7 and MCL1, once it has been phosphorylated, downstream of vincristine treatment [42]. Ubiquitination of phospho-MCL1 by FBW7 led to the destruction of MCL1 by the proteasome. Our recent studies documented MCL1 degradation by vinblastine. We also confirmed that adding vinblastine to GMIC led to disorganization of the microtubule network and cell death. 

IL-17A- and IFN-*γ*-treated DCs also underwent apoptosis upon addition of antibodies neutralizing IL-17A, which selectively reduced BCL2A1 expression. Our interpretation is that the long-term DC survival is dependent on both MCL1 and BCL2A1 expressions. In the future, it would be interesting to evaluate the targeting of both MCL1 and BCL2A1 in chronic IL-17A-related inflammatory diseases, using either Sabutoclax or the combination of toxic compounds targeting MCL1 (such as vinblastin, vincristine, cytarabine, or Obatoclax) with antibodies neutralizing IL-17A, the pro-inflammatory cytokine that induces BCL2A1 in human DC.

## 4. Role of Dendritic Cells and Regulatory T Cells in Rheumatoid Arthritis

**Figure 4:**
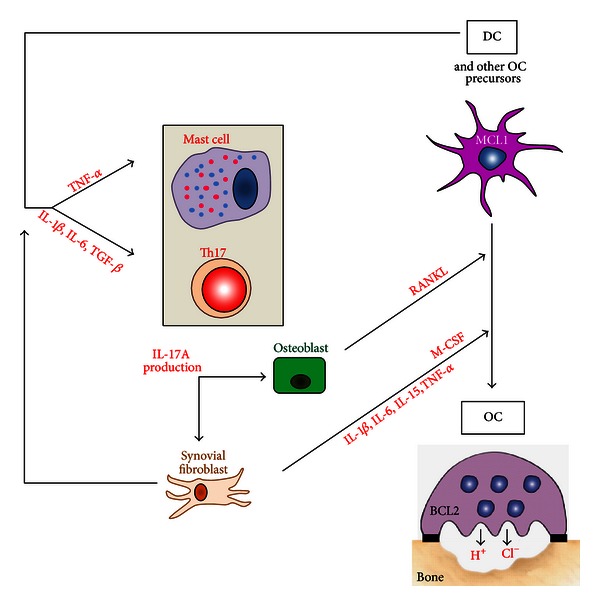
Pro-inflammatory cytokines drive bone resorption in rheumatoid arthritis. Cytokines are amongst the most important mechanisms driving bone resorption associated to inflammation mediated by M-CSF, RANKL, TNF-*α*, IL-1-*β*, IL-6, and finally IL-17A. In RA, IL-17A is mainly produced by Th17 and mastocytes, further amplifying inflammation by enhancing pro-inflammatory cytokine production of synovial fibroblast. IL-17A also increases bone resorption by inducing RANKL production by osteoblasts and M-CSF production by synovial fibroblasts; M-CSF and RANKL are the two cytokines required to differentiate OC from different cell sources: DC, monocytes, macrophages, or bone-marrow progenitors.

RA is a chronic inflammatory disease of the synovium, a delicate membrane that lines the nonweight-bearing surfaces of the joint. In the absence of disease, synoviocytes produce factors that provide nutrition and lubrication for the surrounding cartilage tissue; few cellular infiltrates are seen in the synovium. In RA, the synovium is infiltrated by chronic inflammatory cells, such as macrophages, DC, neutrophils, T cells, and B cells. The resident fibroblasts adopt a quasi-malignant phenotype with upregulation of oncogenes, inhibition of apoptosis, and secretion of cytokines, chemokines, and enzymes that reinforce the inflammation and catalyse joint destruction. The resulting pannus acquires the ability to invade and destroy adjacent articular cartilage. Activation of OC in periarticular bone leads to resorption and erosion, a radiologically detectable hallmark of the disease. Similar processes affect the synovium that lines the tendon sheaths, resulting in tendon weakness and rupture, which are responsible for the characteristic deformities of RA. This is a potentially devastating disease that affects the whole individual, reducing the social contribution, destroying the quality of life, and ultimately shortening the patient's lifespan. Pro-inflammatory cytokines are amongst the most important mechanisms driving this process. In particular, M-CSF, RANKL, TNF-*α*, IL-1, and IL-17A play dominant roles in the pathogenesis of arthritis-associated bone loss (Figure 4). A common first line of treatment is methotrexate monotherapy, while nonresponders are treated with agents neutralizing TNF-*α* activity. Recently, in a phase I clinical study, biotherapy involving neutralization of IL-17A reduced signs and symptoms of RA with no strong adverse effects [43]. 

DCs are key players in RA. Rheumatoid synovium is characterized by accumulation of immature and mature DC subsets perivascularly, in close association with T cells and B-cell follicles [44–46]. Synovial fluid contains significant numbers of myeloid DC compared to blood, suggesting a role for these antigen presenting cells in disease perpetuation [47, 48]. DC may contribute to ongoing inflammation through presentation of autoantigens, as suggested by animal models of autoimmune arthritis [49] or secretion of crucial pro-inflammatory mediators or differentiation into OC [4]. Whether regulatory T-cell (T_REG_) defects are present in patients with RA is not clear. The number of CD4^+^CD25^high^  T_REG_ in the peripheral blood of patients with RA was found to be higher than in healthy individuals in one study, but not in others [50]. DCs show evidence of activation *in vivo*: upregulation of MHC, costimulatory molecules, and expression of NF-*κ*B, RANKL, and RANK [51]. The killing of activated DC and the investigation of tolerance induction by shaping DC plasticity towards tolerogenic DC may possibly give rise to a withdrawal of therapy. 

## 5. Targeting MCL1 and BCL2A1 in Rheumatoid Arthritis

**Figure 5:**
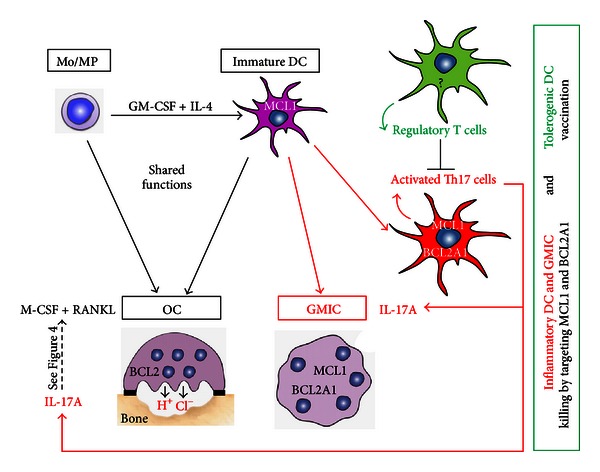
Model for the therapeutical management of diseases associated with IL-17A-dependent chronic inflammation with bone loss. In the context of IL-17A-driven inflammation, IL-17A amplifies OC formation and consequently bone resorption (see details in Figure 4). In addition, IL-17A induces BCL2A1 expression in MCL1^+^ monocyte-derived DC, DC clustering, and fusion, leading to a mixed culture, containing both mononuclear and multinuclear (GMIC) inflammatory DC. These inflammatory DC and GMIC express MCL1 and BCL2A1 contrary to OC whose survival is under the control of BCL2. Tolerogenic DCs activate regulatory T cells able to control activated Th17 cells. The question mark indicates that the status of BCL2 family in tolerogenic DC is unknown and should be studied to validate this model. In order to cure diseases with IL-17A-dependent chronic inflammation associated with bone loss, we suggest killing inflammatory DC and GMIC by targeting both MCL1 and BCL2A1. In parallel performing therapeutic autologous vaccination with tolerogenic DC may help breaking IL-17A-dependent chronic inflammation to restore normal bone physiology.

MCL1 is critical for the survival of macrophages in the joints of patients with RA, thus representing a potential therapeutic target in this disease [52]. In a mouse study, BIM-BH3 mimetic therapy reduced arthritis through the activation of myeloid cell apoptosis, thus demonstrating that BH3 mimetic therapy may have a significant potential for the treatment of RA [53]. More recently, Oliveira et al. evaluated gene expression profiles of (i) RA patient responders and nonresponders to methotrexate and, in the case of nonresponders, (ii) the responders and nonresponders to methotrexate combined with anti-TNF-*α* biotherapy [54]. They identified nine genes in methotrexate nonresponders and three genes in methotrexate plus anti-TNF-*α* nonresponders. Two genes were common in both lists: CCL4 and BCL2A1. This is a strong argument to further evaluate the role of BCL2A1 in RA and, in particular, a potentially overlooked role of long-term surviving IL-17A-stimulated MCL1^+^ BCL2A1^+^-activated DCs. Segura et al. have just characterized that inflammatory DCs, found in human inflammatory fluids, represent a distinct human DC subset, sharing gene signatures with *in vitro* monocyte-derived DC and involved in the induction and maintenance of Th17 cell responses [55]. If the survival pathway of these inflammatory DC is different from that of tolerogenic DC (Figure 5), it would be possible, on the one hand, to vaccinate with autologous DC exhibiting potent tolerogenic functions and, on the other hand, to induce apoptosis of inflammatory DC, in order to reinstate immune tolerance [56] and to abrogate IL-17A-dependent DC-driven inflammation.

## 6. Role of Dendritic Cells and Regulatory T Cells in Langerhans Cell Histiocytosis

LCH is a rare disease which belongs to the histiocytic disorders characterized by tissue damage induced by infiltrating cells (histiocytes), derived from the monocytic lineage [57]. LCH occurs predominantly in children but can occur at any age. Clinical manifestations can vary from a single self-resolving lesion to a severe life-threatening systemic form. Multiple organs may be affected by this disease including bone (80% of the patients), skin, lymph nodes, endocrine glands, and the central nervous system. LCH lesions are heterogeneous and form aggressive granulomas containing CD1a^+^ CD207^+/-^ cells (presumed to be pathogenic LCH cells) admixed with macrophages, T cells, eosinophils, and MGC [58]. Killing the lesional tissue-aggressive LCH cells is difficult but may be achieved in most patients by chemotherapy regimens containing the combination of prednisone and vinblastine or, in salvage settings, cladribine and cytarabine. 

The exact origin of pathogenic LCH cells is unclear. Based on many common features, it was proposed that they arise from epidermal CD207^+^ Langerhans cells. However, in 2008, we proposed that pathogenic DC may derive from monocytes rather than belonging to the Langerhans cell lineage [7]. This is, in keeping with data from a recent gene expression profile study of human cells isolated from LCH granulomas, also suggesting that LCH lesions originate from accumulation of immature myeloid DC rather than epidermal Langerhans cells [59]. Finally, LCH DCs exhibit a unique transcription profile that separates them from all previously known DCs based on their expression of both Notch ligand and its receptor [60].

The etiology of LCH remains controversial between an inflammatory disorder, a neoplasm, or even both since induction of long-term DC survival by inflammation may license accumulation of mutations; this might provide to LCH DC a more apoptotic-resistant behavior. Senechal et al. found that less than 2% of cells were proliferating within lesions and propose that pathogenic DC accumulation is mainly the consequence of increased survival rather than proliferation [61]. This was associated with a local and systemic expansion of CD25^high^  FoxP3^high^  T_REG_ possibly impairing the resolution of LCH granulomas [61]. Altogether, these data suggest that immunological mechanisms play the major role in the development of LCH. Furthermore, evidence of concordance for LCH in monozygous twins supports the concept of a genetic predisposition to develop LCH, possibly affecting the immune system regulation [62]. However, Badalian-Very and colleagues found that 57% (35 of 61) of examined LCH specimens display the oncogenic BRAF V600E mutation [63]. These findings were also confirmed by additional independent studies [64, 65]. BRAF is a pivotal protein kinase of the RAS-RAF-MAPK signaling pathway which regulates cell survival and proliferation. In pathological LCH cells, constitutive activity of the mutant BRAF V600E protein may lead to a deregulated signaling through this pathway, thereby resulting in increased cell survival [66].

## 7. Targeting MCL1 and BCL2A1 in Langerhans Cell Histiocytosis

In LCH lesions, apoptotic pathways have been shown to be active alongside prosurvival pathways [67–69], and the expansion or healing of a granuloma is likely the sum of these apparently conflicting activities. Concerning the BCL2 family members, BCL2 expression was documented in LCH DC in two separate studies using immunohistochemistry and *in situ* hybridization [68, 69]. However, BCL2 was not found to be elevated in CD207^+^ cells from LCH lesions analyzed by transcriptome analysis by Allen et al. [59], who instead showed upregulation of BCL2L1 and BAX. Similar data on BCL2L1 had been previously described in pulmonary LCH [70]. Whether upregulation of survival molecules in LCH is due to exogenous stimuli, such as cytokines, or intrinsic mutations such as BRAF V600E, in the RAS-RAF-MAPK signaling cascade [63] in a majority of samples from LCH biopsies, is still not clarified. So far, no other cancerogenic mutations have been found in LCH and the BRAF V600E mutation by itself is not sufficient for tumor development [71]. Southern blot analysis performed by the Savell team showed no evidence for gene rearrangement of the BCL2 gene [68]. An abundant number of cytokines have been described in LCH lesions, many with the potential to affect cell survival [72, 73]. Considering the presence also of IL-17A in LCH and the therapeutic efficacy of vinblastine, targeting MCL1, it would be interesting to study the role of MCL1 and BCL2A1 in LCH and to correlate their expression to disease progress and drug resistance. Depending on these future studies, targeting MCL1 and BCL2A1 in LCH may be of importance, at least to prevent the intense bone resorption occurring in 80% of the patients with LCH. 

## 8. Exploiting DC Surface Molecules to Specifically Target BCL2A1-Expressing DC

There are a variety of *in vivo* DC-targeting strategies used in different contexts including autoimmune disease therapies [74], vaccine-induced immunity [75], and cancer therapy [76]. Specific targeting of long-term survival BCL2A1^+^ DCs may form a promising therapeutic avenue in inflammatory conditions. Two different strategies can be suggested: inhibition of the intracytoplasmic activity of BCL2A1 or prevention of IL-17A signal transduction in DCs. In the former, a BCL2A1 inhibitory peptide (that should be developed) may be delivered directly to DCs by using a fusion protein built with this peptide and GM-CSF, whose receptor is expressed by BCL2A1^+^ DCs. More specifically, it can be loaded into biodegradable nanoparticles attached to monoclonal antibodies that recognize specific DC surface receptor(s). Since human inflammatory DCs appear most likely to be derived from monocytes *in vivo *[55], a potential target surface receptor is CD209/DC-SIGN, expressed by these cells in tissues and absent on the surface of other DC subpopulations. Specific inhibition of IL-17A signal transduction in DCs may be achieved through an approach which combines anti-DC-SIGN and anti-IL-17A antibodies. This may result in the neutralization of IL-17A and the subsequently induced BCL2A1 expression in DCs.

## 9. Conclusion

DCs are critical regulators of immune responses not only at initiation, but also, as recently demonstrated, for the maintenance of chronic inflammation, especially the IL-17A-driven chronic inflammation [55]. We demonstrated that IL-17A activates long-term survival pathway by inducing BCL2A1 in DC, thus providing a molecular basis for DC maintenance in IL-17A-mediated chronic inflammation. Cytokines and BCL2-related survival pathway may interplay to determine not only myeloid cell accumulation and inflammatory DC maintenance, but also their fusion and final differentiation into either GMIC or OC, the bone-resorbing giant cells (Figure 5). This may affect the clinical course and final long-term outcomes of patients. However, fundamental research is required to solve the question mark: a better understanding of the disease-related alterations in BCL2-related survival pathways and functions of DC might offer the opportunity to design and fine-tune approaches aimed at killing inflammatory DC, while therapeutic vaccination may reintroduce tolerogenic DC. Since chemotherapy remains, at present, the standard of care for LCH, introduction of immune-modulation is highly warranted. Currently available data suggest that manipulation of the BCL2 family (with the decrease of both MCL1 and BCL2A1) in DC, associated with a therapeutic vaccination with autologous tolerogenic DC, might represent a suitable treatment in rheumatoid arthritis and Langerhans cell histiocytosis, possibly leading to a cure.

## Figures and Tables

**Table 1:** Approved Hugo gene nomenclature of the BCL2 family.

Approved symbol	Approved name	Activity	Location	Synonym
BCL2	B-cell CLL/lymphoma 2	Prosurvival	18q21.3	Bcl-2, PPP1R50
BCL2L1	BCL2-like 1	Prosurvival	20q11.21	Bcl-X, bcl-xL, bcl-xS, BCL2L, BCLX, PPP1R52
BCL2L2	BCL2-like 2	Prosurvival	14q11.2-q12	BCL-W, KIAA0271, PPP1R51
MCL1	Myeloid cell leukemia sequence 1 (BCL2-related)	Prosurvival	1q21	BCL2L3, Mcl-1
BCL2A1	BCL2-related protein A1	Prosurvival	15q24.3	ACC-1, ACC-2, BCL2L5, BFL1, GRS
BAX	BCL2-associated X protein	Proapoptotic multidomain	19q13.3-q13.4	BCL2L4
BOK	BCL2-related ovarian killer	Proapoptotic multidomain	2q37.3	BCL2L9, BOKL, MGC4631
BAK1	BCL2-antagonist/killer 1	Proapoptotic multidomain	6p21.31	BAK, BCL2L7
BAD	BCL2-associated agonist of cell death	Proapoptotic BH3-only	11q13.1	BBC2, BCL2L8
BID	BH3 interacting domain death agonist	Proapoptotic BH3-only	22q11.2	—
BCL2L11	BCL2-like 11 (apoptosis facilitator)	Proapoptotic BH3-only	2q13	BIM, BimEL, BimL, BimS, BOD
BMF	BCL2 modifying factor	Proapoptotic BH3-only	15q14	FLJ00065
BIK	BCL2-interacting killer (apoptosis-inducing)	Proapoptotic BH3-only	22q13.31	NBK
HRK	Harakiri, BCL2 interacting protein	Proapoptotic BH3-only	12q24.2	DP5
PMAIP1	Phorbol-12-myristate-13-acetate-induced protein 1	Proapoptotic BH3-only	18q21.32	APR, NOXA
BBC3	BCL2 binding component 3	Proapoptotic BH3-only	19q13.3-q13.4	JFY1, PUMA

## Abbreviations

BCL2: B-cell lymphoma 2
BCL2A1: BCL2-related protein A1
DC: Dendritic cell
GMIC: Giant myeloid inflammatory cells
IFN: Interferon
LCH: Langerhans cell histiocytosis
MCL1: Myeloid cell leukemia sequence 1
MGC: Multinucleated giant cells
OC: Osteoclast
RA: Rheumatoid arthritis
SMI: Small molecules inhibitors
T_REG_: Regulatory T cell.
